# Simvastatin suppresses dexamethasone-induced secretion of plasminogen activator inhibitor-1 in human bone marrow adipocytes

**DOI:** 10.1186/1471-2474-12-82

**Published:** 2011-04-27

**Authors:** Kazutaka Sakamoto, Makoto Osaki, Akira Hozumi, Hisataka Goto, Tatsuya Fukushima, Hideo Baba, Hiroyuki Shindo

**Affiliations:** 1 Department of Orthopaedic Surgery, Graduate School of Biomedical Science, Nagasaki University, 1-7-1 Sakamoto, Nagasaki, Nagasaki 852-8501, Japan

## Abstract

**Background:**

Osteonecrosis of the femoral head is a common complication of high-dose glucocorticoid treatment. Intravascular thrombosis is thought to be associated with the ischemic state of the femoral head. Plasminogen activator inhibitor-1 (PAI-1) is an adipokine, which are physiologically active substances secreted from visceral and subcutaneous adipocytes. PAI-1 suppresses fibrinolysis by binding tissue-type plasminogen activator. Several reports have described the relationship between PAI-1 and steroid-induced osteonecrosis of the femoral head, and the preventive effects of lipid-lowering agents (statins) against steroid-induced osteonecrosis of the femoral head. We previously reported that adipokines and dexamethasone induced PAI-1 secretion from bone marrow adipocytes. The purpose of the present study is to examine the effects of simvastatin on PAI-1 secretion from human bone marrow adipocytes in vitro.

**Methods:**

Primary bone marrow adipocytes were extracted from collagenase-treated bone marrow fluid obtained from the femoral necks of 40 patients (6 men, 34 women; age range, 52-81 years) undergoing hip joint replacement surgery. After suspended culture with or without dexamethasone or simvastatin, PAI-1 mRNA expression was assessed by real-time RT-PCR. Total PAI-1 protein secretion in culture medium was assessed by enzyme-linked immunosorbent assay.

**Results:**

PAI-1 mRNA expression was up-regulated by 388% (*P *= 0.002) with dexamethasone, and down-regulated by 45% (*P *= 0.002) with simvastatin, as compared to control levels. Dexamethasone increased total PAI-1 secretion by 166% (*P *= 0.001) and simvastatin decreased total PAI-1 secretion by 64% (*P *= 0.002). No significant changes were observed in adiponectin mRNA expression and secretion by dexamethasone and simvastatin, while pre-treatment with simvastatin reversed dexamethasone induced PAI-1 secretion by 89%, as compared to control levels.

**Conclusion:**

The present study confirmed the suppressive effects of simvastatin on PAI-1 expression and secretion from bone marrow adipocytes. Furthermore, pre-treatment with simvastatin reversed dexamethasone induced PAI-1 secretion. Simvastatin may thus exhibit preventive effects against steroid-induced osteonecrosis of the femoral head by suppressing PAI-1 secretion.

## Background

Osteonecrosis of the femoral head (ONFH) is a common complication of high-dose glucocorticoid treatment. Steroids, such as glucocorticoids, have diverse activities throughout the body in multiple organs, and numerous disorders have been linked to hypercortisolism. Abnormal lipid and protein metabolism, elevated blood glucose, vessel wall weakening and osteoporosis are some of the side effects attributed to receiving glucocorticoids, while hypercoagulation/hypofibrinolysis [1-3], adipogenesis [4,5], endothelial cell apoptosis [6,7], fat-cell enlargement [8], and increased intraosseous pressure [9] are thought to be associated with steroid-induced ONFH.

Recent reports have shown that visceral adipocytes secrete various physiologically active substances known as adipokines [10,11]. In the bone marrow space, there is a large quantity of mature adipocytes that are possible candidates for adipokine secretion. Considering the enclosed nature of the bone marrow space, intramedullary adipocytes may be involved in bone metabolism, as we previously reported that bone marrow adipocytes express receptor activator of NFκ-B ligand (RANKL) and support osteoclast differentiation [12].

Plasminogen activator inhibitor-1 (PAI-1), one of the adipokines secreted by adipocytes [13,14], suppresses fibrinolysis by binding tissue-type plasminogen activator (t-PA), and a relationship between PAI-1 and thrombosis or hypercoagulation has been suggested. Furthermore, blood coagulation in the femoral head is thought to be associated with osteonecrosis. There are several reports regarding increased PAI-1 secretion in blood sera of patients with ONFH [15-18]. We also previously reported about dexamethasone-induced PAI-1 secretion from human bone marrow adipocytes [19].

There are several studies indicating that statins, 3-hydroxy-3-methylglutaryl coenzyme A (HMG-CoA) reductase inhibitors, have both lipid-lowering effects and preventive effects against steroid-induced ONFH [20-23]. However, the detailed mechanisms of these preventive effects against steroid-induced ONFH are unclear.

The purpose of the present study is to examine the effects of statins on PAI-1 expression and secretion in primary bone marrow adipocytes in vitro.

## Methods

### Study subjects

**Figure 1:**
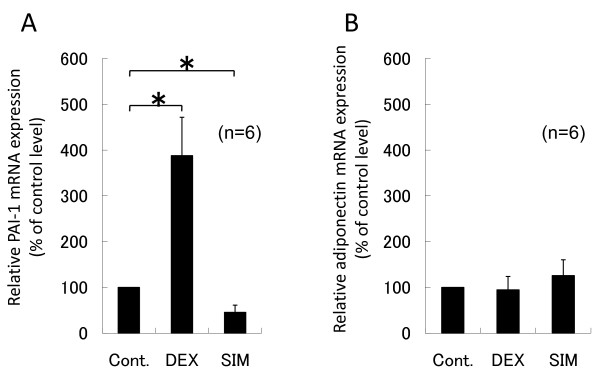
**Effects of dexamethasone and simvastatin on PAI-1 (A) and adiponectin (B) mRNA expression**. Human bone marrow adipocytes were cultured in serum-free DMEM with 1 μM dexamethasone or 10 μM simvastatin for 24 h. PAI-1 and adiponectin mRNAs extracted from the floating adipocyte layer were assessed by real-time RT-PCR. Cont.: control group; DEX: dexamethasone-treated group; SIM: simvastatin-treated group. PAI-1 mRNA expression was up-regulated by dexamethasone and down-regulated by simvastatin. Data represent relative expression levels vs. control levels and are expressed as means ± SD. *P < 0.05 between two values.

**Figure 2:**
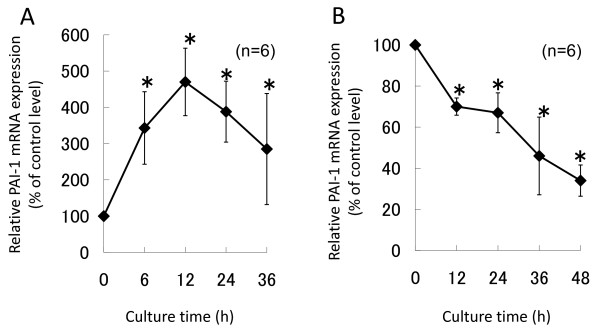
**Time-course study of PAI-1 mRNA expression with dexamethasone or simvastatin**. Human bone marrow adipocytes were cultured in serum-free DMEM with 1 μM dexamethasone (A) or 10 μM simvastatin (B). Total RNA was extracted from the floating adipocyte layer and PAI-1 mRNA were detected by real-time RT-PCR at 6, 12, 24, 36 and 48 h. PAI-1 mRNA expression was significantly up-regulated by dexamethasone at 12 h. PAI-1 mRNA expression was gradually down-regulated by simvastatin. Data represent relative expression levels vs. control levels at identical time points and are expressed as means ± SD. *P < 0.05 vs. untreated control cells at identical time points.

**Figure 3:**
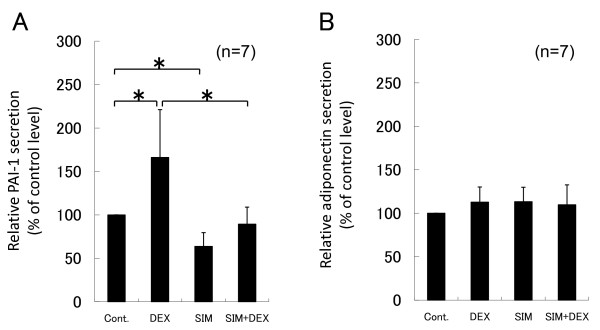
**Effects of dexamethasone and simvastatin on total PAI-1 and adiponectin protein secretions after 36 h of suspension culture**. Human bone marrow adipocytes were cultured in serum-free DMEM with 1 μM dexamethasone, 10 μM simvastatin or 10 μM simvastatin pretreatment following 1 μM dexamethasone at 12 h. ELISA analysis of total PAI-1 protein secretion (A) and adiponectin protein secretion (B) were performed after 36 h of suspension culture. Cont.: control group, DEX: dexamethasone-treated group, SIM: simvastatin-treated group, SIM+DEX: simvastatin following dexamethasone-treated group. Total PAI-1 secretion was up-regulated by dexamethasone and down-regulated by simvastatin. Simvastatin reversed the dexamethasone induced PAI-1 secretion below control levels. Dexamethasone and simvastatin slightly, but not significantly, increased adiponectin secretion. Data represent relative secretion levels vs. control levels and are expressed as means ± SD. *P < 0.05 between two values.

**Figure 4:**
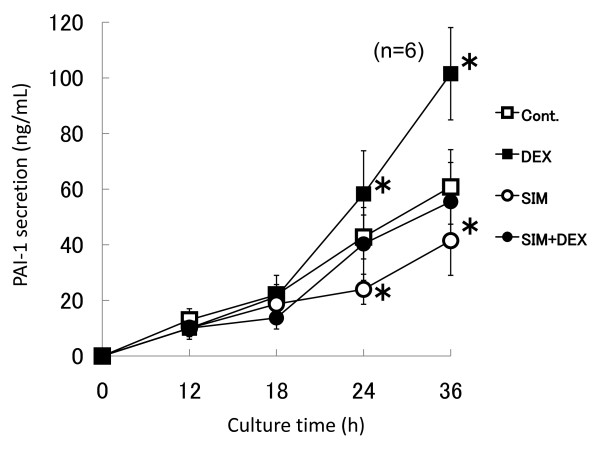
**Time-course study of total PAI-1 protein secretion with dexamethasone and simvastatin**. Human bone marrow adipocytes were cultured in serum-free DMEM, and divided into four groups. Cont.: untreated; DEX: treated with 1 μM dexamethasone at 12 h; SIM: treated with 10 μM simvastatin at 0 h; SIM+DEX: treated with 10 μM simvastatin at 0 h following 1 μM dexamethasone at 12 h. ELISA of total PAI-1 protein secretion in culture medium was performed at 12, 18, 24 and 36 h. Pre-treatment of simvastatin down-regulated total PAI-1 protein secretion induced by dexamethasone to control levels. Data represent total PAI-1 protein secretion levels (ng/mL), and are expressed as means ± SD. *P < 0.05 vs. untreated control cells at identical time points.

Bone marrow fluid was obtained from the femoral necks of 40 patients (6 men, 34 women; age range 52-81 years) undergoing hip joint replacement surgery. Patients with diabetes mellitus, rheumatoid arthritis, and metabolic bone disorders and those with a history of glucocorticoid therapy were excluded. We divided samples into three or four groups, including the control, dexamethasone and simvastatin groups. Each experiment was performed with samples from 6 or 7 patients (n = 6 or 7). The energy status in each experiment is as follow; Figure 1A, B: mean BMI, 24.9; mean age, 69.3 years, Figure 2A, B: mean BMI, 23.3; mean age, 64.8 years, Figure 3A, B: mean BMI, 24.4; mean age, 66.5 years, Figure 4: mean BMI, 23.7; mean age, 60.3 years. BMI and age were analyzed statistically, but no significant trends were noted. The study protocol was approved by the Institutional Ethics Review Board and informed consent was obtained from each patient before surgery.

### Isolation and culture of primary adipose cells

Bone marrow fluid was mixed with 20 mL of Dulbecco's modified Eagle's medium (DMEM: Gibco BRL, Grand Island, NY) and treated with 0.1% collagenase A (Sigma Chemical Co., St. Louis, MO) for 1 h at 37°C. Digested cells were then centrifuged at 200 × g for 5 min, and the adipocyte layer was carefully aspirated from the upper lipid phase [12,19,24]. This procedure includes bone marrow collection and complete cell digestion by collagenase treatment, and because only adipocytes float in the medium, they can be isolated by centrifugation and aspiration of the floating layer. To purify the isolated adipocytes, cells were filtered through a 200-μm diameter nylon mesh, and washed three times with fresh medium. Adipocytes were counted, and 5×10^6 ^cells were then suspended in 2 mL serum-free DMEM in 15-mL Falcon tubes and subjected to suspension culture in the presence of 1 μM dexamethasome (Wako Pure Chemical Industry, Osaka, Japan) or 10 μM simvastatin (Toronto Research Chemicals, Toronto ON, Canada) under 5% CO_2 _at 37°C. Samples of primary adipose cells and conditioned medium were taken at various times over the course of 48 h.

### Analysis of PAI-1 and adiponectin mRNA expression

PAI-1 and adiponectin mRNA expression was measured by quantitative real-time reverse transcription-polymerase chain reaction (RT-PCR). Total RNA was extracted from adipocytes using an RNeasy Mini Kit 250 (Qiagen, Chatsworth, CA) according to the manufacturer's instructions. Total RNA (100 ng) was reverse-transcribed with Taqman One Step PCR Master Mix Reagents kit (Applied Biosystems, Foster City, CA). Sequences of PCR primers were PAI-1 (SERPINE1, Hs00167155_m1), adiponectin (ADIPOQ, Hs00605917_m1), and GAPDH (GAPDH, Hs99999905_m1) (Taqman Gene Expression Assay, Applied Biosystems; product name, product number, respectively). PCR assays were conducted using the ABI PRISM 7000 Sequence Detector System (Applied Biosystems) according to the manufacturer's instructions. Expression of each mRNA was estimated after adjustment for GAPDH mRNA expression.

### Analysis of PAI-1 and adiponectin protein secretion

PAI-1 and adiponectin levels in culture medium were quantified by enzyme-linked immunosorbent assay (ELISA). The protein levels of total PAI-1 and adiponectin were determined using an LPIA-PAI test (Mitsubishi Chemical Yatron, Tokyo, Japan) and a human adiponectin ELISA kit (Otsuka Pharmaceutical Co., Ltd., Tokyo, Japan), respectively.

### Statistical analysis

Results are shown as means ± SD. Data were analyzed using SPSS (Version 16.0). Mann-Whitney test was used to compare each parameter. *P *values of < 0.05 were considered to be statistically significant.

## Results

### PAI-1 and adiponectin mRNA expression regulated by dexamethasone or simvastatin

PAI-1 and adiponectin mRNA expression in bone marrow adipocytes were examined by RT-PCR. PAI-1 mRNA expression was significantly up-regulated by 388% (*P *= 0.002) with dexamethasone and down-regulated by 45% (*P *= 0.002) with simvastatin, as compared to control levels (Figure 1A), while adiponectin mRNA expression was 95% with dexamethasone, and 125% with simvastatin (Figure 1B).

A time-course study of PAI-1 mRNA expression with 1 μM dexamethasone or 10 μM simvastatin was performed. Significant increases in PAI-1 mRNA expression were observed using dexamethasone. Peak PAI-1 mRNA expression was 490% (*P *= 0.001), as compared to control levels, at 12 h (Figure 2A), and was down-regulated by 34% (*P *= 0.001) at 48 h with simvastatin (Figure 2B).

### PAI-1 and adiponectin protein secretion regulated by dexamethasone or simvastatin

Total PAI-1 and adiponectin levels in culture medium were measured by ELISA after 36 h of suspension culture. Dexamethasone increased total PAI-1 secretion by 166% (*P *= 0.001) and simvastatin decreased total PAI-1 secretion by 64% (*P *= 0.002), as compared to control levels, while pre-treatment with simvastatin reversed dexamethasone-induced total PAI-1 secretion by 89% (*P *= 0.109) (Figure 3A). Dexamethasone and simvastatin slightly, but not significantly, increased adiponectin secretion. Pre-treatment with 10 μM simvastatin following 1 μM dexamethasone slightly increased adiponectin secretion by 110% (*P *= 0.249), as compared to control levels (Figure 3B).

A time-course study of total PAI-1 secretion with dexamethasone or simvastatin was conducted and the total PAI-1 in culture medium was measured by ELISA. We divided bone marrow adipocytes into four groups, and total PAI-1 protein secretion was determined by ELISA at 12, 18, 24 and 36 h. The four groups were: untreated controls; treatment with 1 μM dexamethasone at 12 h; treatment with 10 μM simvastatin at 0 h; and treatment with 10 μM simvastatin at 0 h, followed by 1 μM dexamethasone at 12 h. At 24 h and 36 h, dexamethasone increased total PAI-1 secretion by 136% (58.3 ng/mL: *P *= 0.002) and 167% (101.5 ng/mL: *P *= 0.001), respectively, as compared to control levels. At 24 h and 36 h, simvastatin decreased total PAI-1 secretion by 56.1% (24 ng/mL: *P *= 0.002) and 68.3% (41.5 ng/mL: *P *= 0.002), respectively, as compared to control levels. Simvastatin pretreatment following dexamethasone showed no significant changes at 24 h and 36 h (Figure 4).

## Discussion

Steroid-induced ONFH is a severe complication in patients receiving steroid treatment for rheumatoid arthritis, collagen diseases, allergies, or after having undergone transplant surgery. The pathogenesis of ONFH is unclear, but there is now consensus on the etiopathogenesis of ONFH, both in terms of intravascular thrombosis-induced occlusion and extravascular lipid-deposition-induced pressure, which leads to impairment of the intra-osseous blood supply [25]. Glucocorticoid also induces intravascular hypercoagulation and hypofibrinolysis by oxdative stress to endothelial cells. [6,7]. In contrast, lipid transportation to peripheral tissue, elevated adipogenesis and enlargement of adipocytes are involved in extravascular events [4,5,8]. It has been reported that statins prevent the incidence of steroid-induced osteonecrosis and the enlargement of bone marrow adipocytes in rabbits [26].

Recent studies have shown that visceral adipocytes secrete various physiologically active substances, known as adipokines, that play important roles in metabolic syndrome [10,11,13,14]. However, there are few reports on bone marrow adipocytes and bone metabolism. We previously reported that human bone marrow adipocytes express RANKL and support osteoclast differentiation [12], and increased PAI-1 secretion from bone marrow adipocytes induced by dexamethasone [19]. Blood supply to the femoral head is poor because of its anatomic structure. Therefore, the biological environment of the bone marrow in the femoral head may differ from the external environment of the bone. Taking the enclosed bone marrow space into consideration, PAI-1 expression in bone marrow adipocytes may play an important role in the formation of intravenous thrombi.

The present study demonstrated that dexamethasone up-regulates PAI-1 expression, while simvastatin down-regulates it. PAI-1 mRNA expression was significantly enhanced at 12 h and total PAI-1 secretion was continuously increased when cells were treated with dexamethasone. Our results do not contradict previous reports on increased blood PAI-1 levels and ONFH [15-18]. PAI-1 mRNA expression was gradually suppressed to 38% of control levels by 48 h and total PAI-1 secretion was decreased when cells were treated with simvastatin. Pre-treatment with simvastatin reversed dexamethasone-induced PAI-1 protein secretion from bone marrow adipocytes. A hypercoagulated and ischemic state in the femoral head may occur during increased PAI-1 secretion from bone marrow adipocytes. Therefore, suppressing PAI-1 secretion may be important for preventing steroid-induced ONFH. Our findings support previous findings on the protective effects of statins against steroid-induced ONFH [20-23].

Recent reports have shown that statins also have protective effects against atherosclerosis and metabolic syndrome [27-32]. In these reports, statins were shown to suppress PAI-1 mRNA expression in endothelial cells [29-31], visceral and subcutaneous adipocytes [27,28]. PAI-1 mRNA expression is thought to be activated via the type-1 angiotensin II receptor (AT1 receptor) and Rho pathway [33-35]. As statins suppress the production of metabolic products, including geranylgeranyl pyrophosphate (GGPP), from mevalonic acid to cholesterol synthesis, they may inactivate Rho through the suppression of GGPP synthesis and down-regulate PAI-1 mRNA expression. In bone marrow adipocytes, PAI-1 mRNA expression may also be regulated via the AT1 receptor and Rho pathway.

Low levels of hepatic cytochrome P4503A (CYP3A), an enzyme that inactivates steroids, have been reported in patients with ONFH [36,37]. Simvastatin induces CYP3A activity and prevents the development of ONFH in patients receiving steroid treatment [38]. Although we used simvastatin in the present study, it remains uncertain which statin is most effective in suppressing PAI-1 secretion from bone marrow adipocytes.

Adiponectin is an important endocrine factor regulating insulin sensitivity, volume of visceral adipose mass and lypolysis, and is closely associated with whole body metabolism [10,11]. Glucocorticoids induce expression of leptin, particularly in visceral adipocytes obtained from obese individuals [39], whereas adiponectin is down-regulated by dexamethasone [40]. In bone marrow adipocytes, dexamethasone showed no significant effects on adiponectin and TNF-α secretion in a previous study [19]. In the present study, no significant effects on adiponectin expression or secretion were observed after treatment with dexamethasone or simvastatin; however, there may be differences between bone marrow adipocytes and subcutaneous, visceral adipocytes.

## Conclusion

Simvastatin down-regulated PAI-1 mRNA expression and protein secretion in human bone marrow adipocytes. Simvastatin also suppressed dexamethasone-induced PAI-1 secretion in bone marrow adipocytes. Our results may provide insight into one of the mechanisms by which simvastatin prevents steroid-induced ONFH.

## Competing interests

The authors declare that they have no competing interests.

## Authors' contributions

KS and MO carried out PCR and immunoassays, and drafted the manuscript. HB and HS were involved in the conception and design of the study. AH, HG and TF were involved in sample collection during hip surgery and primary adipocyte culture. All authors were involved in drafting manuscript and revising it for critically important content. All authors have read and approved the final manuscript.

## Pre-publication history

The pre-publication history for this paper can be accessed here:

http://www.biomedcentral.com/1471-2474/12/82/prepub
